# Supplementary material for: Identification and functional analysis of non-coding regulatory small RNA FenSr3 in Bacillus amyloliquefaciens LPB-18
Source: PeerJ. 2023 May 15;11:e15236. doi: 10.7717/peerj.15236 (PMC10194069; doi:10.7717/peerj.15236)
Supplement: Supplemental Information 4 [file peerj-11-15236-s004.zip › KO/CK-vs-T1_map/map05163.html]

KEGG PATHWAY: Human cytomegalovirus infection - Reference pathway


|  |  |
| --- | --- |
| **Human cytomegalovirus infection - Reference pathway** |  |

[
Pathway menu
| Organism menu
| Pathway entry
| Show description
| User data mapping
]

Human cytomegalovirus (HCMV) is an enveloped, double-stranded DNA virus that is a member of beta-herpesvirus family. HCMV is best known for causing significant morbidity and mortality in immunocompromised populations. As with other herpesviruses, HCMV gB and gH/gL envelope glycoproteins are essential for virus entry. HCMV gB could activate the PDGFRA, and induce activation of the oncogenic PI3-K/AKT pathway. Though it is unlikely that HCMV by itself can act as an oncogenic factor, HCMV may have an oncomodulatory role, to catalyze an oncogenic process that has already been initiated. US28, one of the four HCMV-encoded vGPCRs (US27, US28, UL33 and UL78), also has a specific role in the oncomodulatory properties. In addition, HCMV has developed numerous mechanisms for manipulating the host immune system. The virally encoded US2, US3, US6 and US11 gene products all interfere with major histocompatibility complex (MHC) class I antigen presentation. HCMV encodes several immediate early (IE) antiapoptotic proteins (IE1, IE2, vMIA and vICA). These proteins might avoid immune clearance of infected tumor cells by cytotoxic lymphocytes and NK cells.

|  |  |  |
| --- | --- | --- |
| Reference pathway | 184% 150% 122% 100% 82% 67% 55% | 图片下载 |
